# Case Report: Hydroa vacciniforme-like lymphoproliferative disorder, an EBV-associated disease, successfully treated with hematopoietic stem cell transplantation

**DOI:** 10.3389/fimmu.2025.1511385

**Published:** 2025-03-21

**Authors:** Eduardo Liquidano-Perez, Gibert Maza-Ramos, Marco Yamazaki-Nakashimada, Rodolfo Rodríguez-Jurado, Alfonso G. Ramírez Ristori, Juan Carlos Bustamante-Ogando, Mario Ernesto Cruz-Munoz, Arturo Gutierrez-Guerrero, Marimar Saez-de-Ocariz, Sara Espinosa-Padilla, Nideshda Ramirez-Uribe, Selma C. Scheffler-Mendoza

**Affiliations:** ^1^ Immunodeficiency Research Laboratory, National Institute of Pediatrics, Mexico City, Mexico; ^2^ Faculty of Medicine, Autonomous University of Guerrero, Acapulco de Juárez, Guerrero, Mexico; ^3^ Department of Pediatric Clinical Immunology, National Institute of Pediatrics, Mexico City, Mexico; ^4^ Pathology Department, National Institute of Pediatrics, Mexico City, Mexico; ^5^ Molecular Immunology Laboratory at the Faculty of Medicine, Autonomous University of Morelos, Cuernavaca, Mexico; ^6^ Department of Dermatology, National Institute of Pediatrics, Mexico City, Mexico; ^7^ Transplant Department, National Institute of Pediatrics, Mexico City, Mexico

**Keywords:** hydroa vacciniforme, T-cell, cutaneous lymphoma, stem cell transplantation, Epstein-Barr virus infections, human herpesvirus, lymphoproliferative disorders, case report

## Abstract

**Introduction:**

The hydroa-vacciniforme-like lymphoproliferative disorder (HVLD) is a rare NK/T-cell condition affecting children in Latin America and Asia. It often progresses to systemic lymphoma, with Latin American patients experiencing worse outcomes compared to East Asians. Understanding viral and host genetic interactions is crucial for advancing targeted therapies. Here, we report a male patient with HVLD successfully treated with hematopoietic stem cell transplantation, highlighting its potential as a therapeutic approach for this aggressive disease.

**Case description:**

An 8-year-old boy presented with persistent skin lesions, fever, and pain. Biopsy confirmed a diagnosis of HVLD. Initial treatments with thalidomide and steroids provided temporary relief. At 12, lymphoma progression led to rituximab and CHOP chemotherapy. Further investigations revealed persistent EBV infection and lymphoma; hence, a haploidentical stem cell transplant was performed at 15. The procedure was successful, achieving complete immune reconstitution and viral clearance. Three years post-transplant, the patient remains in good health with no detectable EBV and complete vaccinations.

**Discussion:**

While EBV infection is common, only specific immunodeficiency states seem to enable EBV-related lymphoproliferative disorders. The exact mechanism leading to this immunosuppressive environment in HVLD remains unclear. Clinically, HVLD resembles specific inborn errors of immunity with EBV susceptibility. Additionally, cases of GATA2 and TACI deficiency presenting with HVLD suggest a potential link to underlying immune dysfunction. Further research in this area is crucial to understand the immunological basis of HVLD. Treatment options for HVLD are diverse and lack standardized protocols. Our case demonstrates the potential of HSCT with reduced-intensity conditioning and EBV-specific T-cell infusion as an effective cure. Given the limited understanding of HVLD, an immunological approach to characterizing patient profiles and prolonged follow-up are essential. While diverse therapies exist, HSCT offers the best hope for a cure. Further research towards tailored treatment strategies holds significant promise for improved patient outcomes.

**Conclusion:**

HVLD presents a complex and multifaceted challenge; our case demonstrates the potential of HSCT as a curative treatment. Unveiling the underlying immunology and tailoring therapies to patient profiles are crucial for improved outcomes. Further research is key to refining treatment strategies and offering hope for this rare and severe disease.

## 1 Introduction

Epstein–Barr virus (EBV) is a ubiquitous human herpesvirus that maintains lifelong subclinical persistent infections in susceptible humans. It has been associated to a tumor-inducing human virus related with epithelial cell carcinomas and lymphomas (1). Despite the high prevalence of EBV infection, only 1%–2% of all human malignancies are associated with this virus. Most of the people control persistent EBV infection with an immune mechanism mediated by cytotoxic lymphocytes, mainly NK, γδ, and CD8+ T cells (2).

The discovery and detailed investigation of inborn errors of immunity (IEI) characterized by heightened susceptibility to EBV-induced disease have revealed several cell types and signaling pathways that play critical and non-redundant roles in the host defense against EBV. These analyses have demonstrated mechanisms underlying EBV-induced disease in rare genetic conditions, identified molecules and pathways that could be targeted to treat severe EBV infection, and potentially enhance the efficacy of an EBV-specific vaccine (3, 4).

The hydroa vacciniforme-like lymphoproliferative disorder (HVLD) is a rare, chronic, and aggressive lymphoproliferative disorder associated to NK and/or T cells. This disorder has been associated with EBV infection and can be aggravated over the years, sometimes evolving into systemic lymphoma and generally with a poor prognosis. It affects mostly children and adolescents in Latin America and Asia. Most of the reported Latin American patients usually have an aggressive and fatal disease, whereas most patients in East Asia survive longer. So far, there is no standardized treatment. We present an HVLD-affected male successfully treated with hematopoietic stem cell transplantation (HSCT) (5–7).

## 2 Case description

**Figure 1:**
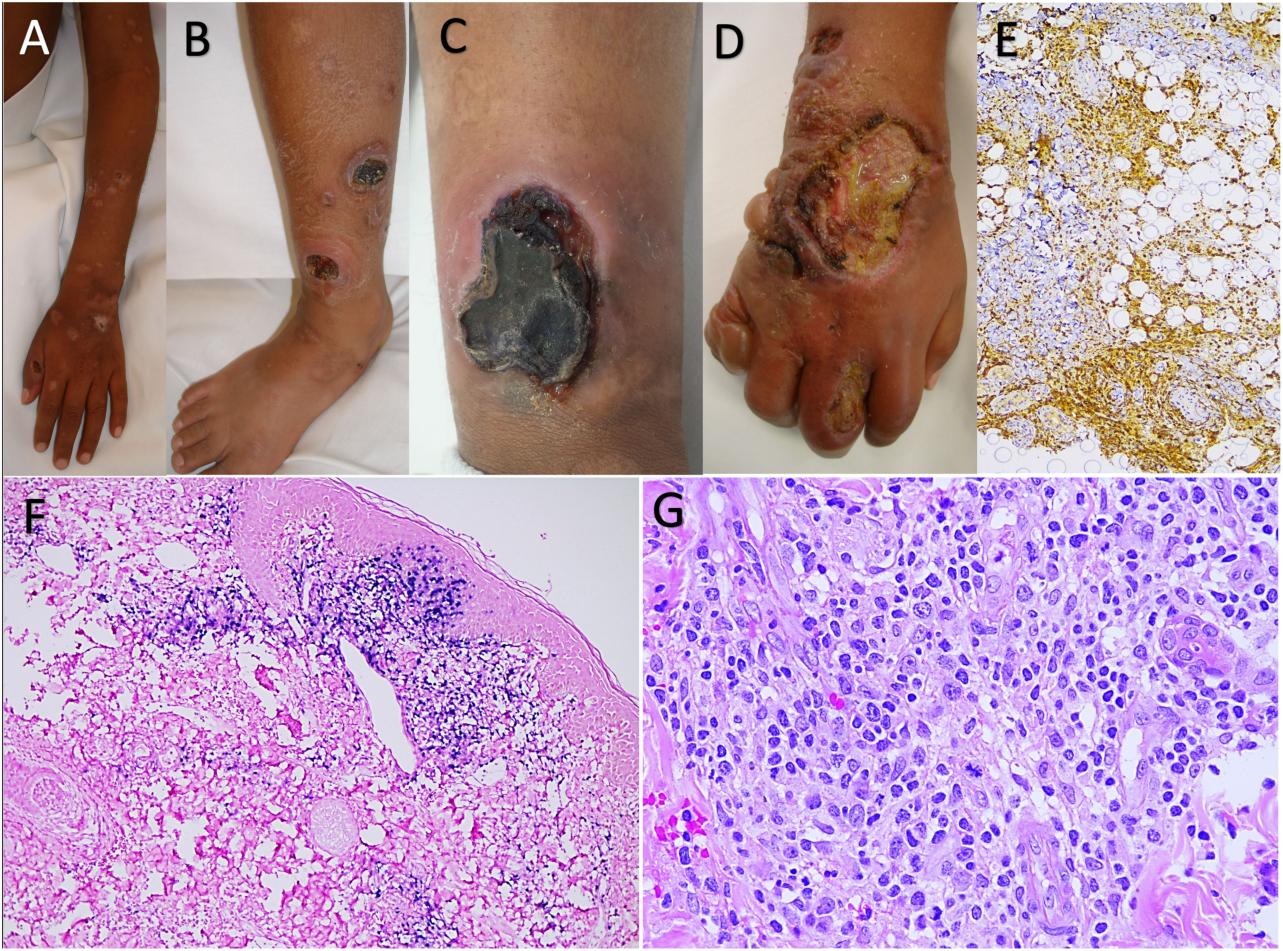
**(A)** Ulcers, residual hyperpigmented spots, and atrophic scars larger than 5 mm in diameter. **(B)** Leg edema associated with eschars, ulcers, varioliform scars, and hyperpigmented spots. **(C)** Ulcer with elevated, erythematous-violaceous, and ill-defined edges associated with perilesional edema. **(D)** Edematous right hand with edema, erythema, vesicles, blisters, and ulcers with a dirty bottom and well-defined edges, progressing to atrophic, depressed, and varioliform aspects. **(E)** Immunohistochemical reaction (immunoperoxidase method) with antibodies for CD3+ and CD8+; the inflammatory infiltrate is cytotoxic T immunophenotype (CD8+). **(F)** Chromogenic *in situ* hybridization result for Epstein–Barr virus (EBER), which is positive in atypical T lymphocytes of the subepidermal inflammatory infiltrate. **(G)** The lymphocytes of the inflammatory infiltrate show marked atypia, with large, hyperchromatic nuclei, and some vesicular with poorly distributed clumpy chromatin; they present notches around their contour and visible nucleoli. Atypical mitotic figures are observed.

A 4-year-old boy, with no relevant family history, started with intermittent fever; 6 months later, he developed erythematous macules that evolved into papules, vesicles, blisters, ulcers, and scars, healing with hyperpigmented spots affecting sun-exposed and covered areas (**Figures 1A–C**). He received multiple topical treatments with temporary and partial improvement.

At 8 years, he was examined at our center, on physical examination showed erythematous plaques 1.5–2 cm in diameter with central necrosis and 1 cm ulcers with erythematous border covered by central crust, multiple atrophic scars, and residual hyperpigmented spots. He presented with fever and incapacitating, constant pain that did not improve with analgesics. The skin biopsy showed CD8+ lymphocytic infiltrate from the superficial dermis to the adipose tissue with angiocentricity and epidermotropism, confirming a diagnosis of HVLD (**Figure 1E**). He initially received management with thalidomide (100 mg/day) and persisted with intermittent exacerbations; therefore, steroids and antibiotics were intermittently added.

At 12 years, he relapsed despite treatment, and cutaneous lesions progressed and were more aggressive (**Figure 1D**); a new biopsy showed cutaneous T-cell lymphoma (**Figures 1F, G**). The whole blood EBV-DNA viral load was 1.5 × 10^6^ copies, so four doses of rituximab (375 mg/m2bs/dose) were given with a significant reduction in the viral load.

Due to the diagnosis of lymphoma, he received primary treatment with prednisone, intravenous gamma globulin (IVIG) (1 g/kg/month), hydroxychloroquine (200 mg/day), vitamin D (3200 UI/day), and R-CHOP. However, the hepatosplenomegaly did not improve, the skin ulcers improved partially with the appearance of new lesions, and the viral load persisted high, so eight additional doses of R-CHOP were given to reduce the viral load.

Immunological studies showed the following: IgG, 2,040 mg/dl (650–1,210); IgM, 54.1 mg/dl (130–370); IgA, 192 mg/dl (70–220); lymphocytes CD3+, 2,516 cells/mm^3^ (1,200–2,600); CD4+, 695 cells/mm^3^ (650–1,500); CD8+, 1,755 cells/mm^3^ (370–1,100); CD20+, 530 cells/mm^3^ (270–870); CD56+, 530 cells/mm^3^ (100–480).

**Figure 2:**
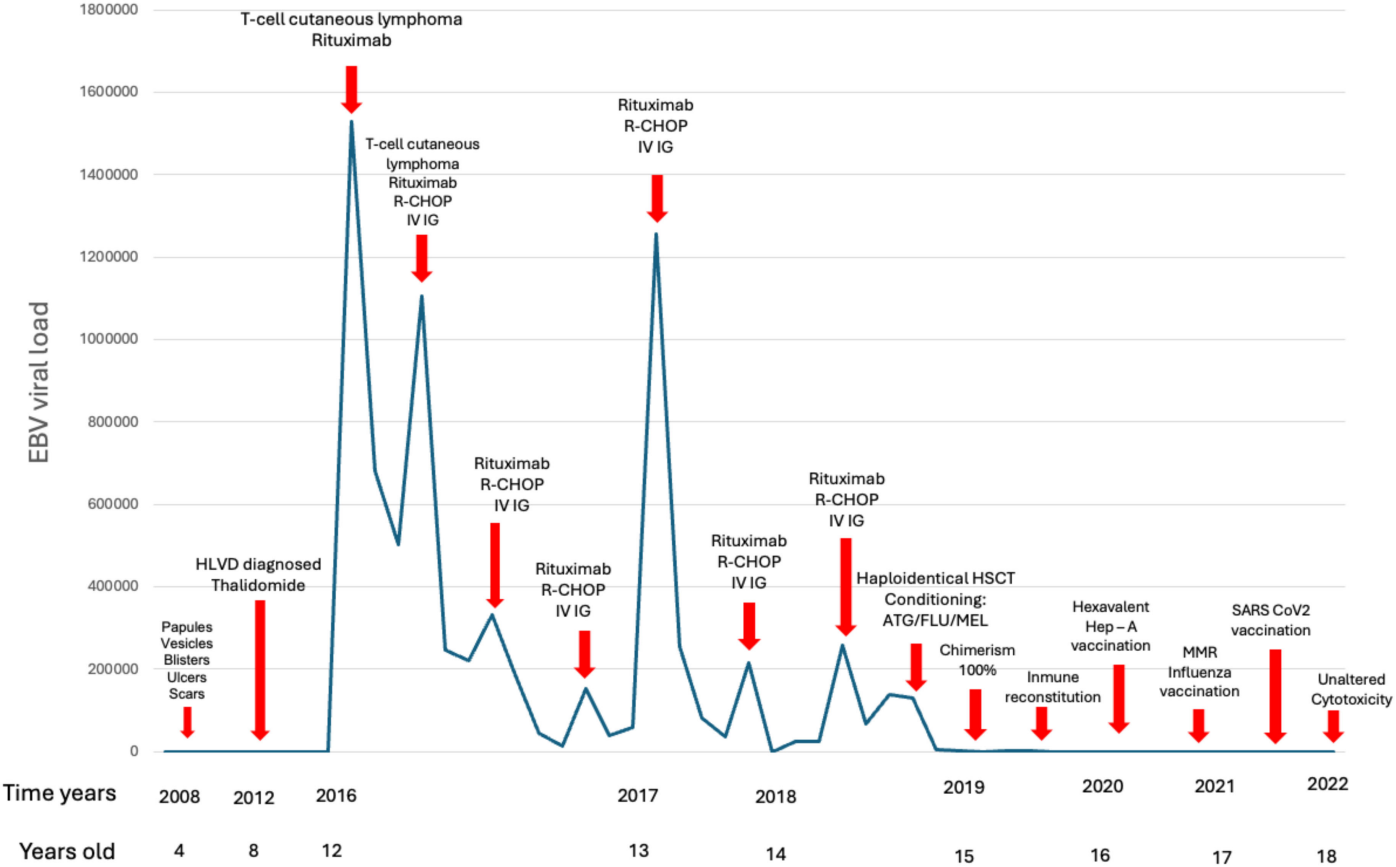
Time graph where the X-axis represents the year and age of the patient against the Y-axis with the EBV viral load. The treatments used are indicated with arrows. EBV viral load was not tested from 2008 to 2012. Once immunological reconstitution is achieved, control of the chronic EBV infection is achieved. Completing a catch-up in the vaccination scheme, including immunization against SARS Cov2.

Owing to the diagnosis with persistent and chronic EBV infection and a malignant lymphoproliferative disorder, we decided to perform HSCT to achieve immunological reconstitution and exert a graft-versus-tumor effect. Consequently, a haploidentical stem cell transplantation with cyclophosphamide post-infusion was carried out from a 15-year-old sister donor. He received reduced intensity conditioning (antithymocyte gamma globulin cumulative dose, 5 mg/kg; fludarabine, 30 mg/m^2^/day/5 days, melphalan 100 mg/day/2 days), and a total dose of 5 × 10^6^ CD34+ cells/kg from peripheral blood was infused. Graft-versus-host-disease (GVHD) prophylaxis was based on mycophenolate and tacrolimus. HSCT was successful with a chimerism of 100% at day +180. Complete immune reconstitution was achieved at 6 months: CD3+, 1,603 cells/mm^3^; CD4+, 514 cells/mm^3^; CD8+, 1,089 cells/mm^3^; CD20+, 1,361 cells/mm^3^; CD56+, 30 cells/mm^3^; IgA, 67 mg/dl; IgG, 1,263 mg/dl; and IgM, 66 mg/dl on day +376. After 3 years of post-HSCT, he is in good general condition, has negative EBV viral loads, and with a complete revaccination scheme, including two doses against SARS CoV2 (**Figure 2**). The patient was transferred to an adult care unit, reporting improvement in his quality of life and satisfaction with the results of the interventions applied.

## 3 Discussion

In 1995, Ruiz-Maldonado et al. described a multisystemic disorder, which was named at the time “edematous, scarring vasculitic panniculitis,” emphasizing to be a distinct disease from hydroa vacciniforme. Fever, cutaneous affection, hepatosplenomegaly, and cytopenia with malignant potential were prominent features (8). With time, the term HVLD was coined. HVLD is considered a rare entity, although more than 160 cases have been described in the past 31 years. All the cases were either Latin American or Asian patients; given the ubiquity of EBV infection, this geographical variation suggests that regional factors such as dietary habits, socioeconomic stratum, and ethnic distributions influence the carcinogenesis associated with EBV (9). It also has been described that genome sequence polymorphism in the EBV in T or NK cell diseases reflects the geographic origins of the patients and not a distinct type of EBV (10). Another plausible marker of racial susceptibility is the HLA complex, which is involved in the immunological regulation of viral infections (11). Unfortunately, there are no descriptions of HLA haplotype expression for HVLD; therefore, further studies are needed to prove HVLD susceptibility (1).

The skin biopsy exhibits EBER-positive and LMPO-negative lymphocytes. Moreover, in electron microscopy, EBV-DNA is found in lymphocytes and intralesional keratinocytes (12). Skin samples analyzed by whole exome sequencing found somatic gene variants in five genes: STAT3, ELF3, KMT2D, CHD7, and IKBKB (13).

HVLD gather clinically many features found in IEI: susceptibility to EBV infection, autoimmune features with severe vasculitis, and susceptibility to develop lymphoma. However, a monogenic defect in this disease has not been found.

The immunological features of these patients represent a window of opportunity for research in this field; a limitation of our case is that the curative treatment was given to the patient before an extensive genetic test was done. Interestingly, two known IEI have presented with HVLD. A 24-year-old Cantonese woman with EBV hydroa vacciniforme-like lymphoma, disseminated *Mycobacterium avium* complex, and hemophagocytic lymphohistiocytosis who had GATA2 deficiency (14). Grześk et al. present a case of a 14-year-old boy with CMV and EBV infection without HLH who was diagnosed through next-generation sequencing with a mutation in the TNFRSF13B gene, which caused a defective production of the TACI protein confirming the diagnosis of the heterozygous variant of CVID (15). Indeed, HVLD is not listed in the IEI; however, the inability to control the infection, impaired immune surveillance against EBV, and these two cases add evidence to consider a possible underlying immunological defect in these patients.

The therapeutic options in the patient with HVLD are not well established, since there are no controlled clinical trials. There are diverse treatments proposed in several published cases including systemic corticosteroids, chemotherapy, thalidomide, methotrexate, interferon, hydroxychloroquine, IVIG, and HSCT. There are some successful cases treated with these different therapeutic options. Transfer of donor EBV-specific cytotoxic T cells may be beneficial in these cases. A retrospective review of 10 patients with HVLD treated with IVIG showed a mean survival of 0.88 months (mean follow-up of 38 months) with a reduction in the rate of hospitalizations and use of antibiotics, so that IVIG may be a cost-effective intervention as a bridging therapeutic option before HSCT (16). The isolated use of rituximab to reduce the viral load of EBV is not effective for T- and NK-associated EBV, so in this patient, it was used in association with CHOP (R-CHOP), considering that the patient was suffering from lymphoma. Other alternatives for the treatment of EBV-associated lymphoma are SMILE and DA-EPOCH (17). Guo et al. report 19 patients with HVLD, and only one was alive, the one who received allogeneic transplantation (18). HSCT offers the only established curative option; however, it should be kept in mind that chronic EBV infection is a condition associated with treatment failure as described by Saldaña and Yi (19, 20). There are a small number of reported cases of HSCT in HVLD, from which we have learned:

1. Reduced-intensity conditioning reduces transplant-related mortality (21, 22).
2. The infusion of EBV-specific lymphocytes from the donor is beneficial (21, 22).
3. Both sources, cord blood, and bone marrow may be equally effective as sources for a successful HSCT. However, there are differences regarding the outcome since engraftment failure is slightly higher in cord blood transplants, and the ratio of severe acute GVHD might be more significant in bone marrow transplants (22).

Finally, we consider that patients with HVLD need an immunological approach to understand the immunological profile of these group of patients; also we recommend a prolonged follow-up even if the disease is in remission. We suggest performing an HSCT as the only curative option.

## Data Availability

The data underlying this study are not publicly available due to patient privacy issues. The data are available from the corresponding author upon reasonable request.
